# Does distribution of menstrual products through community-based, peer-led sexual and reproductive health services increase use of appropriate menstrual products? Findings from the Yathu Yathu trial

**DOI:** 10.1186/s12978-023-01631-x

**Published:** 2023-06-20

**Authors:** Bernadette Hensen, Melleh Gondwe, Mwelwa Phiri, Ab Schaap, Lucheka Sigande, Sian Floyd, Melvin Simuyaba, Rosemary Zulu-Phiri, Louis Mwape, Sarah Fidler, Richard Hayes, Musonda Simwinga, Helen Ayles

**Affiliations:** 1 Sexual and Reproductive Health Group, Department of Public Health, Institute of Tropical Medicine, Nationalestraat 155, 2000 Antwerp, Belgium; 2 Clinical Research Department, London School of Hygiene and Tropical Medicine, London, UK; 3 Zambart, Lusaka, Zambia; 4 Department of Infectious Disease Epidemiology, London School of Hygiene and Tropical Medicine, London, UK; 5 Imperial College and Imperial College NIHR BRC, London, UK

## Abstract

**Background:**

Globally, millions of adolescent girls and young women (AGYW) who menstruate have limited access to appropriate and comfortable products to manage their menstruation. Yathu Yathu was a cluster randomised trial (CRT) that estimated the impact of community-based, peer-led sexual and reproductive health (SRH) services on knowledge of HIV status among adolescents and young people aged 15–24 (AYP). Among the services offered through Yathu Yathu were free disposable pads and menstrual cups. This study aimed to investigate whether the availability of free menstrual products through Yathu Yathu increased AGYW’s use of an appropriate menstrual product at their last menstruation and explored the characteristics of AGYW who accessed menstrual products through Yathu Yathu.

**Methods:**

Yathu Yathu was conducted between 2019 and 2021 in 20 zones across two urban communities of Lusaka, Zambia. Zones were randomly allocated to the intervention or standard-of-care arm. In intervention zones, a community-based hub, staffed by peers, was established to provide SRH services. In 2019, a census was conducted in all zones; all consenting AYP aged 15–24 were given a Yathu Yathu Prevention Points Card, which allowed AYP to accrue points for accessing services at the hub and health facility (intervention arm), or the health facility only (control arm). Points could be exchanged for rewards, thus acting as an incentive in both arms. We conducted a cross-sectional survey in 2021 to estimate the impact of Yathu Yathu on the primary outcome (knowledge of HIV status) and secondary outcomes. Sampling was stratified by sex and age group; we analysed data from AGYW only to estimate the impact of Yathu Yathu on use of an appropriate menstrual product (disposable or reusable pad, cup, tampon) at last menstruation. We analysed data at zone-level using a two-stage process recommended for CRTs with < 15 clusters/arm.

**Results:**

Among 985 AGYW participating in the survey who had experienced menarche, the most commonly used products were disposable pads (88.8%; n = 875/985). At their last menstruation, 93.3% (n = 459/492) of AGYW in the intervention arm used an appropriate menstrual product compared to 85.7% (n = 420/490) in the control arm (adjPR = 1.09 95%CI 1.02, 1.17; p = 0.02). There was no evidence for interaction by age (p = 0.20), but use of appropriate products was higher among adolescents in the intervention arm relative to control (95.5% vs 84.5%, adjPR = 1.14 95%CI 1.04, 1.25; p = 0.006) with no evidence for a difference among young women (91.1% vs 87.0%, adjPR = 1.06 95%CI 0.96, 1.16, p = 0.22).

**Conclusions:**

Delivering community-based peer-led SRH services increased the use of appropriate menstrual products among adolescent girls aged 15–19 at the start of the Yathu Yathu study. With less economic independence, the free provision of appropriate menstrual products is critical for adolescent girls to access materials that allow them to effectively manage their menstruation.

**Supplementary Information:**

The online version contains supplementary material available at 10.1186/s12978-023-01631-x.

## Introduction

Globally, millions of adolescent girls and young women (AGYW) who menstruate have limited access to appropriate and comfortable products to manage their menstruation. For many, the cost of appropriate menstrual products poses the greatest barrier to use, while access to water and sanitation and disposal facilities, which also help to manage menstruation, may be difficult or constrained [1]. The use of relatively ineffective and non-absorbent materials, such as cloths or tissue paper, may cause discomfort and may be associated with an increased risk of urogenital infections, including bacterial vaginosis and vulvovaginal candidiasis [2, 3]. Furthermore, inadequate management of menstruation has implications for AGYW’s participation in social, educational, and economic activities for, among other reasons, fear of leaks and stains and the stigma associated with menstruation [4] .

Improving access to appropriate menstrual products is one requirement to achieving menstrual health, defined as “…a state of complete physical, mental, and social well-being and not merely the absence of disease or infirmity, in relation to the menstrual cycle.”[5] Over the past decade, inadequate access to menstrual products has gained prominence as an issue of global concern [6]. In Zimbabwe, a qualitative study reported that some AGYW aged 16–24 consider menstruation a “burden” as they are unable to meet the costs associated with accessing products and analgesics to manage the pain of menstruation [7]. Both have an impact on school attendance [7, 8], and evidence suggests that some AGYW sell sex to support meeting the costs of these essential products [7, 9].

Achieving menstrual health requires strategies that deliver menstrual products for free or at low-cost to AGYW from locations acceptable and accessible to them. One strategy is to distribute free products in schools, as has been promised and/or implemented by the governments of several countries, including Kenya, New Zealand and Uganda [10]; or additionally through community centres, youth clubs and pharmacies as in Scotland [11]. Cluster randomised trials (CRT) conducted in Kenya have shown that distribution of pads and/or cups in schools increases AGYW’s comfort during menstruation[9] and general self-efficacy, but found no evidence that this increased school attendance [12]. One CRT found that distribution of pads and cups was associated with a lower risk of sexually transmitted infections, with the authors hypothesising that this reduced risk was driven by a reduced reliance on transactional sex to afford menstrual products; the distribution of cups was also associated with reduced prevalence of bacterial vaginosis [12]. Although school-based delivery is critical to ensuring adolescents have access to appropriate menstrual products, reaching AGYW not in school and offering AGYW greater confidentiality in accessing menstrual products requires distribution through other community-based spaces.

There is limited research on AGYW’s access to menstrual products in Zambia. Qualitative research highlights that some AGYW use inappropriate materials, including cloths and cotton, to manage their menstruation, being unable to afford disposable pads or appropriate reusable products [13, 14]. This research has also found that girls miss school due to limited access to appropriate products [13]. Furthermore, and as has been reported in other countries [4, 15], menstruation is stigmatised; the use of non-absorbent materials raises fears of inadvertent disclosure that one is menstruating by stains on clothes [13, 14, 16, 17]. To avoid this disclosure, and the possibility of being mocked and teased, girls miss school and/or isolate themselves socially [13, 17]. These studies highlight that, in Zambia, a barrier to AGYW’s ability to manage their menstruation is their limited access to appropriate menstrual products. Distribution of free menstrual products from places accessible and acceptable to AGYW would contribute to meeting the menstrual health needs of AGYW in Zambia.

Yathu Yathu was a cluster randomised trial (CRT) that estimated the impact of community-based, peer-led sexual and reproductive health (SRH) services on knowledge of HIV status and various secondary outcomes. Among the services offered through the Yathu Yathu intervention were free disposable pads and menstrual cups. Research conducted while the trial was ongoing showed that Yathu Yathu was an important source of menstrual products, particularly for adolescent girls aged 15–19 [17], and that cups were acceptable and preferred by some AGYW over pads [18]. Using data collected during an endline cross-sectional survey to estimate the impact of Yathu Yathu on the primary and secondary outcomes of the CRT, we investigated whether distribution of menstrual products through community-based peer-led spaces, which offered comprehensive SRH services, increased AGYW’s use of an appropriate menstrual product at their last menstruation. We also explored whether there was evidence of a difference in met menstrual needs and the characteristics of AGYW who accessed menstrual products through Yathu Yathu.

## Methods

### Study population and location

The Yathu Yathu CRT was conducted in two urban communities of Lusaka, Zambia. Each community was divided into 10 zones of approximately equal population size (~ 2350 AYP per zone). As described in detail elsewhere [19], in each community, zones were randomly allocated, in a 1:1 ratio, to the Yathu Yathu intervention or control arm. Prior to the implementation of the Yathu Yathu intervention, all households in both trial arms were enumerated and all AYP aged 15–24 were offered a prevention points card (PPC). The PPC aimed to incentivise service use, as described below, and allowed service access to be monitored by the study team.

### The Yathu Yathu strategy

Yathu Yathu was implemented between 2019 and 2021. The strategy consisted of community-based delivery of comprehensive SRH services, including HIV testing, contraceptives, and menstrual products (free disposable pads and the menstrual cup). Services were delivered by peer support workers at spaces (hubs) in a central location in each zone that was randomly allocated to the intervention arm (n = 10 hubs). Peer support workers managed the day-to-day activities at the hubs and provided information to AYP, either one-to-one or through comprehensive sexuality education sessions. When accessing services, AYP could collect points for services accessed. The numbers of points redeemable for the services accessed were dependent on the anticipated psychological difficulty of accessing individual services. For menstrual products, collecting pads was initially redeemable at 65 points but, after adaptations to the intervention design, no points were redeemable for collecting pads as it was found that accessing pads from hubs posed little to no psychological difficultly for AGYW [17, 20]. Two packs of disposable pads could be collected twice per month.

Menstrual cups were donated to Yathu Yathu by *Chicashana,* a Zambian menstrual health company that aims to provide solutions to menstrual health issues, particularly in rural Zambia. Six-hundred cups were donated, with some used for demonstrations; to motivate access, AGYW could gain 500 points for collecting a cup, with a limit of one cup per AGYW [18]. AGYW accessing cups were given information on how to use cups from peer support workers [18].

Menstrual products were also available for “purchase” as rewards (reusable pads, tampons and branded disposable pads) using points AGYW accrued for accessing services, in addition to other rewards such as soap, toothbrush, toothpaste. Although one-point was equivalent to 0.05 Kwacha (USD 0.002), to facilitate access to better quality and reusable menstrual products, the menstrual products that were available as rewards could be accessed for fewer points than their market value (Table 1). AGYW could obtain reward products as frequently as they wanted, if they had sufficient points available.

**Table 1:** Menstrual products available through Yathu Yathu as a (free) service or reward and the points associated with these products

Service Products	Number of points accrued when accessing the product
Collection of disposable sanitary pads (before adaptations*)	65
Menstrual cup (can only collect once)	500
Collection of disposable sanitary pads (after adaptations)	0

Reward Products	Number of points required to redeem the product
Branded disposable pads (before adaptations*)	380
Branded disposable pads (after adaptations)	140
Pack of branded disposable pads and a razor (before adaptations*)	513
Pack of branded disposable pads and a razor (after adaptations)	297
Branded tampons (before adaptations*)	560
Branded tampons (after adaptations)	140
Reusable sanitary pads	1500

*Before adaptations—the pilot implementation phase, between Sept 2019 and Feb 2020, before changes were made to the system based on lessons learnt from this pilot implementation

### Data sources

We used two data sources for this analysis. The first was data from the endline cross-sectional survey, conducted in 2021, which measured the impact of Yathu Yathu on the primary and secondary outcomes of the CRT [19, 21]. The questionnaire used in this endline survey measured socio-demographics, access to HIV-related services, and use of menstrual products at last menstruation. The sample size, to measure the primary and secondary outcomes, was 2000 AYP, with equal numbers of AYP in four age/sex groups (15–19 and 20–24, male and female). The second data source was the routinely collected PPC data, which included data on the number of visits to the hubs and on the services and rewards accessed during hub visits.

### Outcomes of interest and explanatory variables

The main outcome of interest was use of an appropriate menstrual product (defined as use of a disposable/reusable pad, menstrual cup or tampon) at last menstruation, as self-reported in the endline cross sectional survey among AGYW in the intervention and control arms. We also explored responses to four questions from the Menstrual Practice Needs Scale (MPNS36) [22]. Only four questions were included as menstrual experience was not a predefined outcome of the Yathu Yathu CRT and adding all 36 questions would have increased the time commitment required from study participants. The four questions focussed on material needs at last menstruation, namely: *my menstrual materials were comfortable; I had enough of my menstrual materials to change them as often as I wanted to; I was satisfied with the cleanliness of my menstrual materials,* and *I could get more of my menstrual materials when I needed to*, as we assumed Yathu Yathu might improve access to appropriate menstrual products. Response options were never, sometimes (less than half the time), often (more than half the time), and always [22].

Using the PPC data, the outcome of interest was ever accessing menstrual products from Yathu Yathu, among AGYW who participated in the endline cross-sectional survey and were resident in a zone that received the intervention. The PPC data were linked to the survey data by use of the unique PPC number. Explanatory factors, measured in the survey and explored for their association with access to menstrual products through Yathu Yathu included: age (at time of distribution of the PPC), educational attainment, whether AGYW were currently in school, currently married and employed, and whether, at any point in the last four weeks, there was no food in their household due to lack of resources to access food. This last variable was explored as, with reference to findings emerging from qualitative research conducted by the study team [17], it was assumed that AGYW with less disposable income at household-level would be more likely to access products from the hubs.

### Data analysis

To estimate the impact of Yathu Yathu on use of an appropriate menstrual product and the four material needs questions, we first described these outcomes. For the four needs questions, we described the responses to the original four categories. Next, we created a binary variable for each question, coding Always as 1 and all other responses as 0. We then compared the five outcomes across the two trial arms. With 10 zones per arm, we used a “two-stage” cluster-level analysis appropriate for CRT with < 15 clusters/arm [23].

First, for each zone, we estimated the proportion of AGYW using an appropriate menstrual product and the proportion responding always to each of the four material needs questions. We then calculated the average value of the cluster-specific proportions. Next, we calculated the prevalence ratio (PR) comparing the Yathu Yathu arm with the control arm. To formally compare the trial arms in an “unadjusted” analysis, we fitted a linear regression model of log(cluster-level proportion) on community and trial arm to obtain a log(PR) comparing the Yathu Yathu with the control arm and estimated the corresponding 95% confidence interval (95%CI). To obtain an estimate adjusted for age and education, we followed the 2-stage procedure [23]. In Stage 1, a logistic regression model, which included community, age group and educational attainment, was fitted to the individual-level data. Using this model, we predicted the individual probability of each outcome, under the null hypothesis of no effect of the Yathu Yathu intervention. To estimate cluster-specific expected numbers of individuals with each outcome under the null hypothesis of no effect of Yathu Yathu, we aggregated these individual-level probabilities by cluster. In Stage 2, for each cluster, we calculated the ratio of the observed (O) to the expected (E) number of individuals with the outcome (O/E) and calculated the log [ratio-residual; log (O/E)]. We then fitted a linear regression model of log (O/E) on trial arm and community, to obtain an adjusted PR and corresponding 95%CI. Using logistic regression, we explored whether, at the individual-level, responding always to the four menstrual needs questions was associated with use of an appropriate product at last menstruation.

To explore factors associated with ever accessing menstrual products through Yathu Yathu, we restricted analyses to AGYW resident in intervention zones. We coded AGYW as 1 if they ever collected any menstrual products through Yathu Yathu, according to the PPC data, including menstrual products available for “purchase” using points (i.e. reward menstrual products), and 0 otherwise. We used logistic regression, with a fixed effect to account for differences between enumeration zones and adjusted for age at enumeration, to assess the association between the explanatory variables and ever accessing menstrual products. Variables associated with accessing products at the p < 0.10-level in the age-adjusted analysis were included in adjusted analyses.

## Results

Overall, 996 AGYW participated in the endline survey. The characteristics of AGYW were balanced across the trial arms; half the participants were aged 15–19, approximately 30% were currently in school and one-third reported being married/cohabiting. However, more AGYW in the control arm reported having attained complete secondary or higher education than AGYW in the intervention arm (33.5% n = 166, vs 23.6%, n = 118, respectively; Table 2).

**Table 2:** Key demographic characteristics of the adolescent girls and young women participating in the endline survey, by arm, 2021

	Yathu Yathu Arm (N = 500)		Control Arm (N = 496)	
	Number	Percent	Number	Percent
Age				
15–19	252	50.4	249	50.2
20–24	248	49.6	247	49.8
Highest level of education				
None/ incomplete primary	27	5.4	26	5.2
Complete primary	51	10.2	42	8.5
Incomplete secondary	283	56.6	249	50.2
Complete secondary	118	23.6	166	33.5
Higher education	21	4.2	13	2.6
Currently in school				
No	346	69.2	365	73.6
Yes	154	30.8	131	26.4
Currently employed				
No	405	81.0	409	82.5
Yes	95	19.0	87	17.5
In last 4 weeks, ever a lack of resources in the household to buy food				
No	347	69.4	316	63.7
Yes	153	30.6	180	36.3
Marital status				
Never married	334	66.8	335	67.5
Married and/or cohabiting	160	32.0	155	31.3
Divorced/ separated/widowed	6	1.2	6	1.2

### Use of an appropriate menstrual product at last menstruation

Of the 996 AGYW, 11 had not yet experienced menarche; 979 AGYW reported their age at first menarche. Among these AGYW, the median age of menarche was 14 (IQR 13–15) years in both arms. When asked what products AGYW mainly used during their menstruation, 89.9% (n = 885/985) reported mainly using appropriate menstrual products, among whom 16.4% (n = 145) reported mainly using appropriate products in combination with rags, cotton, or toilet paper. Among all AGYW, the most commonly used products to manage menstruation were disposable pads (88.8%; n = 875/985), old cloths/rags (13.5%, n = 133/985) and new cloths/rags (6.4%, n = 63/985). Only 0.5% (n = 5/985) reported mostly using a menstrual cup and 1% (n = 11/985) tampons.

At their last menstruation, 89.5% (n = 879/982) of AGYW self-reported using an appropriate product to manage their menstruation. By arm, 93.3% (n = 459/492) of AGYW in the intervention arm used an appropriate menstrual product at last menstruation compared to 85.7% (n = 420/490) in the control arm. After adjustment for educational attainment, which showed some imbalance by arm, there was evidence that use of an appropriate product at last menstruation was higher in the intervention than control arm (adjPR = 1.09 95%CI 1.02, 1.17; p = 0.02; Table 3). By age group, although there was no evidence for interaction (p = 0.20), use of an appropriate product was higher among adolescents aged 15–19 in the intervention arm compared to control (95.5% vs 84.5%, respectively, adjPR = 1.15 95%CI 1.05, 1.25; p = 0.005). There was no evidence for a difference among young women aged 20–24 (91.1% vs 87.0%, respectively, adjPR = 1.06 95%CI 0.96, 1.16, p = 0.22).

**Table 3:** Self-reported use of an appropriate menstrual products at last menstruation among adolescent girls and young women aged 15–24, by trial arm, 2021

	Yathu Yathu Arm	Control Arm	Unadjusted PR	95% CI	Adjusted PR^x^	95% CI	p-value
	93.3	85.5	1.09	1.02, 1.17	1.10	1.02, 1.17	0.02
Adolescent girls (aged 15–19*)	95.5	84.5	1.14	1.05, 1.25	1.15	1.05, 1.25	0.005
Women (aged 20–24*)	91.1	87.0	1.05	0.95, 1.17	1.06	0.96, 1.17	0.22

*PR* prevalence ratio, *95%CI* 95% confidence interval

*Age at time of consent to receive a Yathu Yathu prevention points card, ^x^Adjusted for community, age and education

### Met need for menstrual products

Across both arms, fewer than 5% of AGYW responded “never” to each of the four questions on menstrual needs (Additional file 1: Table S1). Consistently more AGYW in the intervention arm responded “always” to the menstrual needs questions, but there was no statistical evidence of a difference in responses to these four questions (Table 4). The percentages of AGYW who self-reported that, at their last menstruation: their menstrual products were always comfortable (68.9% vs 58.9%, in intervention and control arm, respectively), they were satisfied with the cleanliness of their products (72.2% vs 63.4%, respectively), they always had enough product (64.5% vs 56.9%, respectively) and could get more products if needed (63.6% vs 57.3%, respectively) were consistently around 5–10% higher in the intervention than in the control arm; however, in one zone, only between 2% (n = 1) and 4% (n = 2) of AGYW responded “always” to these four questions. Excluding this zone from analyses, the mean percentage was similar across arms and there was less variation in responses (Additional file 1: Table S2). Results were similar by age group (Additional file 1: Table S3).

**Table 4:** Adolescent girls and young women aged 15–24 responding “always” to questions on met material needs at last menstruation, by arm, 2021

At last menstruation:	Yathu Yathu Arm	Control Arm	Adjusted PR	p-value
	Cluster-level mean % (n/N)	Cluster-level mean % (n/N)	(95%CI)*	
My menstrual materials were always comfortable	68.9 (339/493)	58.9 (290/492)	1.31 (0.67, 2.57)	0.40
Cluster-level range in outcome (%)	(16.0–95.8)	(4.1–95.8)		
I was always satisfied with cleanliness of materials	72.2 (355/493)	63.4 (312/492)	1.31 (0.69, 2.51)	0.39
Cluster-level range in outcome (%)	(26.0–97.9)	(4.1–95.8)		
I always had enough materials to change as often as I wanted	64.5 (318/493)	56.9 (280/492)	1.36 (0.62, 2.95)	0.42
Cluster-level range in outcome (%)	(18.0–100)	(2.0–95.8)		
I could always get more materials when I needed to	63.6 (313/493)	57.3 (282/492)	1.26 (0.67, 2.38)	0.45
Cluster-level range in outcome (%)	(20.0–97.9)	(4.1–95.8)		

*Adjusted for age, community and educational attainment, and accounting for clustering by zone, comparing Always response (coded 1) to all other responses (coded 0)

At individual-level, responding “always” to the four questions on met need for products was associated with use of an appropriate menstrual product at last menstruation (Additional file 1: Table S4). Among AGYW who used an appropriate product at their last menstruation, 72.1% (n = 634) were always satisfied with the cleanliness of their product compared to 27.9% (n = 245) of AGYW who had not used an appropriate product (adjOR = 5.50 95%CI 3.49, 8.66; p < 0.001). Sixty-four percent (63.8% (n = 561)) of AGYW who used an appropriate product at last menstruation reported always feeling able to get more products when needed compared to only 36.2% (n = 318) of AGYW who had not used an appropriate product at last menstruation (adjOR = 3.58 95%CI 2.29, 5.59; p < 0.001).

### Individual-level factors associated with accessing menstrual products through Yathu Yathu

In the Yathu Yathu intervention arm, 83.2% (n = 410/493) of AGYW who experienced menarche self-reported ever attending a hub. According to the PPC data, 76.7% (n = 378) of these AGYW had ever collected menstrual products through Yathu Yathu, among whom 88.4% (n = 334/378) had ever collected freely available disposable pads (Table 5).

**Table 5:** Factors associated with accessing menstrual products through Yathu Yathu among adolescent girls and young women residing in Yathu Yathu intervention clusters (N = 493), 2021

	Number of AGYW (column %)	Number ever-collecting menstrual products (row %)	Crude OR (95% CI)*	Adjusted OR (95%CI)	p-value
Age					
15–19	247 (50.1)	202 (81.8)	1.0	1.0	0.007
20–24	246 (49.9)	176 (71.5)	0.55 (0.35, 0.85)	0.56 (0.35, 0.88)	
Highest level of education					
None/ incomplete primary	27 (5.5)	16 (59.3)	0.29 (0.12, 0.70)	0.29 (0.12, 0.70)	0.01
Complete primary	50 (10.1)	44 (88.0)	1.71 (0.67, 4.35)	1.71 (0.67, 4.35)	
Incomplete secondary	280 (56.8)	221 (78.9)	1.0	1.0	
Complete secondary/higher education	136 (27.6)	97 (71.3)	0.69 (0.42, 1.13)	0.69 (0.42, 1.13)	
Currently in school					
No	343 (69.6)	252 (73.5)	1.0	1.0	0.12
Yes	150 (30.4)	126 (84.0)	1.63 (0.94, 2.84)	1.57 (0.89, 2.76)	
Currently employed					
No	399 (80.9)	311 (77.9)	1.0	1.0	0.79
Yes	94 (19.1)	67 (71.3)	0.88 (0.51, 1.52)	0.93 (0.52, 1.62)	
Marital status					
Never married	327 (66.3)	261 (79.8)	1.0	1.0	0.13
Married and/or cohabiting	160 (32.5)	113 (70.6)	0.71 (0.43, 1.16)	0.58 (0.34, 0.99)	
Divorced/ separated/widowed	6 (1.2)	4 (66.7)	0.51 (0.09, 2.97)	0.51 (0.08, 3.22)	
In last 4 weeks, ever no food in household because lack of resources to buy food					
No	342 (69.4)	254 (74.3)	1.0	1.0	0.30
Yes	151 (30.6)	124 (82.1)	1.36 (0.80, 2.30)	1.33 (0.77, 2.29)	

*OR* odds ratio, *AGYW* adolescent girls and young women aged 15–24, *Adjusted for age and accounting for clustering by zone

In our risk factor analysis, there was strong evidence that age was associated with accessing menstrual products, with 80.6% (n = 202/247) adolescents aged 15–19 accessing pads at the hubs compared to 71.0% (n = 176/246) women aged 20–24 (adjOR = 0.56 95%CI 0.35, 0.88; p = 0.007, comparing 20–24 with 15–19 year olds). Educational attainment was also associated with ever accessing menstrual products; 59.3% (n = 16/27) of the relatively small number of AGYW with no or incomplete primary education ever accessed products compared to 78.9% (n = 221/280) of AGYW with incomplete secondary education (adjOR = 0.29 95%CI 0.12, 0.70; p = 0.01). More AGYW who reported that their household lacked resources to buy food in the last four weeks accessed products from the hubs compared to AGYW whose household did not lack resources (82.1% vs 74.3%, respectively), although there was no statistical evidence of a difference in access (adjOR = 1.33 95%CI 0.77, 2.29; p = 0.30). There was little evidence that other factors were associated with accessing menstrual products.

## Discussion

Our analysis provides evidence that the provision of free menstrual products through community-based, peer-led hubs increased AGYW’s use of appropriate products to manage their menstruation, particularly among adolescent girls aged 15–19, among whom there was almost universal use (96%) of an appropriate material at last period in the Yathu Yathu arm. Despite this impact, use of an appropriate product at last menstruation was high in the control arm, with 85% of adolescents self-reporting use of an appropriate product. Almost one-third of AGYW in both arms were not always satisfied with the comfort of their menstrual materials, did not always have enough material, and did not always feel they could get more materials when needed. In the Yathu Yathu arm, the proportion who accessed menstrual products through Yathu Yathu was higher among adolescents aged 15–19 years (82%) than among 20–24-year-olds (72%). Provision of free menstrual products through easily accessible community-based spaces may be particularly valuable for supporting adolescent girls to achieve good menstrual health.

The availability of free menstrual products through Yathu Yathu led to almost universal use of an appropriate menstrual product at last menstruation among adolescent girls who were aged 15–19 at the time of the Yathu Yathu census. To reduce the burden of menstruation, governments need to develop and implement policies that require free provision of appropriate menstrual products [24], particularly to adolescent girls who are less likely to have the financial means to purchase such products. Although only one country (Scotland) provides menstrual products free to all women, in 2004, Kenya removed taxes on menstrual products and, in 2019, South Africa provided products for free in schools [10]. In addition to the provision of materials, Yathu Yathu peer support workers provided information and support to AGYW. Guidance from UNICEF outlines social support and knowledge and skills as two of four key pillars to effective menstrual health programming [25]. The delivery of menstrual products combined with information by peers likely supported AGYW to access products and use them correctly, as highlighted in our previous research [17]. Programmes and policies to improve menstrual health should consider the valuable role that peers could take towards achieving menstrual health among AGYW.

Despite increased use of appropriate materials, we found no evidence that Yathu Yathu had an impact on comfort of and satisfaction with menstrual products; nor on having enough product or feeling able to access more product when needed. Lack of statistical evidence of impact might reflect the relatively high use of an appropriate material at last menstruation in the control zones, similar to a feasibility study in Uganda, which found high self-reported use of disposable pads among school-going adolescent girls [26]. Use of inappropriate materials can cause leaks and stains, therefore, use of inappropriate materials can lead to fear of inadvertent disclosure of menstruation and teasing, thus to shame and isolation [13, 27]. Despite a lack of evidence for an impact on these material needs questions in cluster-level analysis, we found evidence at the individual-level that use of an appropriate menstrual material at last menstruation was associated with the four material need questions. Due to the cross-sectional nature of our data, we cannot elucidate the direction of causation. However, we expect that use of an appropriate product at last menstruation affected met needs; using an appropriate product likely influences whether AGYW always felt comfortable and were always satisfied with the cleanliness of their menstrual products at last menstruation. AGYW have a right to access products that allow them to manage their menstruation effectively, with evidence that use of absorbent materials increases AGYW’s general self-efficacy [9] and affects their school attendance [28]. In addition to access to free menstrual products and information on how to use them, meeting the menstrual health needs of AGYW requires continued efforts to provide adequate water, sanitation and appropriate disposal facilities, and for there to be locations where AGYW can enact various practices during menstruation in private [22, 29].

The majority of AGYW in the intervention arm had accessed menstrual products at least once from peer-led Yathu Yathu hubs. Our risk factor analysis found that adolescent girls aged 15–19 years were more likely to access menstrual products from the hubs than young women aged 20–24 years, with evidence of lower uptake among the relatively small number of AGYW with no or incomplete primary education. This latter finding may reflect the inverse-equity hypothesis, which posits that more educated and wealthier individuals, generally those with less need for new interventions, are initially more likely to benefit from them, thus creating greater inequities in the short term [30]. Strategies to reach adolescent girls with less access to economic resources and AGYW with no or incomplete primary education are required to achieve equitable access. Qualitative research in Malawi has shown that the cost of menstrual products is inhibitive for girls [28]; similarly, previous qualitative research conducted by our study team showed that hub closures in response to COVID-19 led to “hoarding” of pads once hubs reopened, and that adolescent girls’ access to menstrual products was particularly affected by hub closures [17].

Although not initially planned as a key Yathu Yathu service, the provision of free menstrual products proved critical to encouraging AGYW to access the hubs and to take up other SRH services. These findings are consistent with a study in Zimbabwe, in which free provision of menstrual products and analgesics through integrated SRH services was acceptable to AGYW aged 16–24, and AGYW appreciated the opportunity of accessing these products alongside SRH services [7]. Similarly, our research has shown that integration of menstrual and SRH services, including HIV services, through community-based platforms is feasible and, as evidenced by high uptake of menstrual products at the hubs, acceptable to AGYW. Considering the synergies between menstrual health and SRH, including evidence of an increased risk of infections such as bacterial vaginosis associated with use of inappropriate menstrual materials[2] and the role of hormonal contraceptives in alleviating menstrual symptoms, integration of menstrual and SRH services should be considered a priority [3].

Our study has limitations. As the hubs provided menstrual products, but did not address other domains relevant to menstrual health, for example improving sanitation and disposal facilities, we limited the number of questions on menstrual health in the endline survey. We therefore included four questions on menstrual materials that we considered reflected domains that Yathu Yathu could influence. However, the MPNS36 includes 36 questions. We were therefore limited in our ability to measure met needs [22]. Furthermore, comfort and satisfaction are influenced by access to appropriate sanitation and disposal facilities, which are equally important in achieving improved menstrual health. Our data arise from a cross-sectional survey on last menstruation, with data collected soon after the third wave of COVID-19 in Zambia. Access to appropriate materials in both arms may have been affected by measures in place to control the spread of COVID-19. Had we done our survey at a different time, we may have seen less of an impact on use of an appropriate material among adolescents. Finally, use of an appropriate material at last menstruation was self-reported. Considering the stigma surrounding menstruation [4], including that menstruating is unclean [31], AGYW may have overreported use of an appropriate material at last menstruation [27]. Despite limitations, our data arise from a rigorous CRT of a peer-led strategy to deliver SRH services and demonstrates the feasibility of integrating these services with menstrual health services and of delivering services during a pandemic. In support of scale-up of a Yathu Yathu-type approach, there is a need to understand the cost and the potential economies of scale of the strategy.

Supporting AGYW to achieve menstrual health with dignity is fundamental to human rights [32]. Although achieving menstrual health requires access to more than menstrual products, including social support, access to adequate water and sanitation and disposal facilities [5, 32], provision of free or low-cost menstrual products remains a key requirement to improving menstrual health. With evidence that Yathu Yathu increases use of appropriate products at last menstruation, alongside evidence of increased knowledge of HIV status [21], the strategy provides a unique opportunity to reach AGYW with a range of menstrual products and SRH services. As a peer-led approach, the strategy also provides an opportunity for social support, knowledge, and skills, which are important contributors to improved menstrual health [25]. In settings where AGYW have inadequate access to menstrual products and HIV prevalence is high, the Yathu Yathu strategy should be considered to address the synergistic menstrual, SRH, and HIV prevention health needs of adolescents [3].

## Supplementary Information

**Additional file 1. Table S1.** Four category responses to menstrual material needs questions among adolescent girls and young women aged 15-24, by arm, 2021. **Table S2.** Adolescent girls and young women aged 15-24 responding “always” to questions on met material needs at last menstruation, by arm, 2021 – exclusion of one cluster. **Table S3. **AGYW responding “always” to questions on met material needs at last menstruation, by arm and age group, 2021. **Table S4. **Association between responding “always” to questions on met material needs at last menstruation and self-reporting use of an appropriate menstrual product at last menstruation among adolescent girls and young women aged 15-24 years, 2021

## Data Availability

Data will be available upon reasonable request from the London School of Hygiene and Tropical Medicine.
