# Supplementary material for: Does distribution of menstrual products through community-based, peer-led sexual and reproductive health services increase use of appropriate menstrual products? Findings from the Yathu Yathu trial
Source: Reprod Health. 2023 Jun 20;20:92. doi: 10.1186/s12978-023-01631-x (PMC10283167; doi:10.1186/s12978-023-01631-x)
Supplement: Supplementary file 1 — Additional file 1. Table S1. Four category responses to menstrual material needs questions among adolescent girls and young women aged 15-24, by arm, 2021. Table S2. Adolescent girls and young women aged 15-24 responding “always” to questions on met material needs at last menstruation, by arm, 2021 – exclusion of one cluster. Table S3. AGYW responding “always” to questions on met material needs at last menstruation, by arm and age group, 2021. Table S4. Association between responding “always” to questions on met material needs at last menstruation and self-reporting use of an appropriate menstrual product at last menstruation among adolescent girls and young women aged 15-24 years, 2021 [file 12978_2023_1631_MOESM1_ESM.docx]

**Additional file 1**

**Additional Table S1. Four category responses to menstrual material needs questions among adolescent girls and young women aged 15-24, by arm, 2021**

|  | Yathu Yathu Arm | Control Arm |
| --- | --- | --- |
|  | Number (column %) | Number (column %) |
| Menstrual materials were comfortable |  |  |
| Never | 17 (3.5) | 20 (4.1) |
| Sometimes | 62 (12.6) | 87 (17.7) |
| Often | 75 (15.2) | 95 (19.3) |
| Always | 339 (68.8) | 290 (58.9) |
| Was satisfied with cleanliness of materials |  |  |
| Never | 20 (4.1) | 19 (3.9) |
| Sometimes | 49 (9.9) | 77 (15.7) |
| Often | 69 (14.0) | 84 (17.1) |
| Always | 355 (72.0) | 312 (63.4) |
| Had enough materials to change as often as wanted |  |  |
| Never | 15 (3.0) | 21 (4.3) |
| Sometimes | 76 (15.4) | 105 (21.3) |
| Often | 84 (17.0) | 86 (17.5) |
| Always | 318 (64.5) | 280 (56.9) |
| Could get more materials when needed |  |  |
| Never | 22 (4.5) | 21 (4.3) |
| Sometimes | 73 (14.8) | 91 (18.5) |
| Often | 85 (17.2) | 98 (19.9) |
| Always | 313 (63.5) | 282 (57.3) |

**Additional Table S2. Adolescent girls and young women aged 15-24 responding “always” to questions on met material needs at last menstruation, by arm, 2021 – exclusion of one cluster**

|  | Yathu Yathu Arm | Control Arm |
| --- | --- | --- |
| At last menstruation: | Cluster-level mean % (n/N) | Cluster-level mean % (n/N) |
| Menstrual materials were always comfortable | 68.9 (339/493) | 65.0 (288/443) |
| Cluster-level range in outcome (%) | (16.0-95.8) | (46.9-95.8) |
| Always satisfied with cleanliness of materials | 72.2 (355/493) | 70.0 (310/443) |
| Cluster-level range in outcome (%) | (26.0-97.9) | (46.9-95.8) |
| Always had enough materials to change as often as wanted | 64.5 (318/493) | 63.0 (279/443) |
| Cluster-level range in outcome (%) | (18.0-100) | (46.9-95.8) |
| Could always get more materials when needed | 63.6 (313/493) | 63.2 (280/443) |
| Cluster-level range in outcome (%) | (20.0-97.9) | (42.9-95.8) |

**Additional table S3. AGYW responding “always” to questions on met material needs at last menstruation, by arm and age group, 2021**

|  | Yathu Yathu Arm | Control Arm | Adjusted PR | p-value |
| --- | --- | --- | --- | --- |
| At last menstruation: | Cluster-level mean % (n/N) | Cluster-level mean % (n/N) | (95%CI)* |  |
| Menstrual materials were always comfortable |  |  |  |  |
| Adolescents aged 15-19 | 69.3 (171/247) | 60.0 (148/245) | 1.23 (0.59, 2.59) | 0.56 |
| Women aged 20-24 | 68.5 (168/246) | 57.9 (142/247) | 1.37 (0.71, 2.62) | 0.33 |
| Always had enough materials to change as often as wanted |  |  |  |  |
| Adolescents aged 15-19 | 64.6 (159/247) | 55.0 (136/245) | 1.39 (0.64, 2.99) | 0.38 |
| Women aged 20-24 | 64.9 (159/246) | 58.8 (144/247) | 1.25 (0.64, 2.44) | 0.50 |
| Always satisfied with cleanliness of materials |  |  |  |  |
| Adolescents aged 15-19 | 74.5 (184/247) | 62.4 (154/245) | 1.48 (0.69, 3.19) | 0.30 |
| Women aged 20-24 | 69.7 (171/246) | 64.4 (158/247) | 1.17 (0.68, 2.03) | 0.54 |
| Could always get more materials when needed |  |  |  |  |
| Adolescents aged 15-19 | 64.9 (160/247) | 54.6 (135/245) | 1.44 (0.69, 3.02) | 0.31 |
| Women aged 20-24 | 62.2 (153/246) | 60.0 (147/247) | 1.12 (0.65, 1.91) | 0.67 |

*Adjusted for community and educational attainment, and accounting for clustering by zone, comparing Always response (coded 1) to all other responses (coded 0).

**Additional table S4. Association between responding “always” to questions on met material needs at last menstruation and self-reporting use of an appropriate menstrual product at last menstruation among adolescent girls and young women aged 15-24 years (N=982), 2021**

|  | Number  (column %) | Menstrual materials were always comfortable  (Number (row %)) | Always satisfied with cleanliness of materials (Number (row %)) | Always enough materials to change as often as wanted (Number (row %)) | Could always get more materials when needed (Number (row %)) |
| --- | --- | --- | --- | --- | --- |
| Used appropriate product at last menstruation | |  |  |  |  |
| No | 103 (10.5) | 22 (21.4) | 31 (30.1) | 34 (33.0) | 32 (31.1) |
| Yes | 879 (89.5) | 605 (68.8) | 634 (72.1) | 563 (64.1) | 561 (63.8) |
| Adjusted OR (95%CI)* | - | 7.28 (4.41, 11.98) | 5.50 (3.49, 8.66) | 3.23 (2.08, 5.02) | 3.58 (2.29, 5.59) |
| p-value | - | <0.001 | <0.001 | <0.001 | <0.001 |

*Adjusted odds ratio (OR) adjusted for age, education and trial arm.
